# Supplementary material for: Mechanism of SARS-CoV-2 Nucleocapsid Protein Phosphorylation-Induced Functional Switch
Source: Viruses. 2026 Jan 13;18(1):105. doi: 10.3390/v18010105 (PMC12846656; doi:10.3390/v18010105)
Supplement: Supplementary file 1 [file viruses-18-00105-s001.zip › viruses-4017541-supplementary.pdf]

Supplementary Information

Table S1. PCR primers

Primer name	Sequence	PCR Template
SL1-4_FWD	5′-GAAAT*TAATACGACTCACTATAG*GGAAACC-3′	pIDTSmart-HH-SARS-CoV-2 5′ UTR-FokI (linearized)
SL1-4_REV	5′-mCAGTAATTAGTTATTAATTATACTGCGTGAGTG-3′	
SL5_FWD	5′-GAAAT*TAATACGACTCACTATAG*GGTCGTTGACAGGACACGAG-3′	pIDTSmart-HH-SARS-CoV-2 5′ UTR-FokI (linearized)
SL5_REV	5′-mGTTTTCTCGTTGAAACCAGGGAC-3′	
3D1_FT	5′-CAGAAGGGGATAGAGGCGGCGATCAAGCCTCTGATCGTTCCTCATCACGTAGTCGCAAC-3′	pET28α-10xHis-TEV-SARS-CoV-2 WTNp
3D1_FS	5′-TCATCACGTAGTCGCAAC-3′	
3D1_RT	5′-GGAACGATCAGAGGCTTGATCGCCGCCTCTATCCCCTTCTGCGTAGAAGCCTTTTGGC-3′	
3D1_RS	5′-CGTAGAAGCCTTTTGGC-3′	
3D2/6D_FT (make 3D2)	5′-GAAATTCAGATCCAGGCAGCGATAGGGGAACTGATCCTGCTAGAATGGCTGGCAATGGCG-3′	pET28α-10xHis-TEV-SARS-CoV-2 WTNp
3D2/6D_FS (make 3D2)	5′-GAATGGCTGGCAATGGCG-3′	
3D2/6D_RT (make 3D2)	5′-TAGCAGGATCAGTTCCCCTATCGCTGCCTGGATCTGAATTTCTTGAACTGTTGCGACTAC-3′	
3D2/6D_RS (make 3D2)	5′-TTGAACTGTTGCGACTAC-3′	
3D2/6D_FT (make 6D)	5′- GAAATTCAGATCCAGGCAGCGATAGGGGAACTGATCCTGCTAGAATGGCTGGCAATGGCG -3′	pET28α-10xHis-TEV-SARS-CoV-2 3D1 Np
3D2/6D_FS (make 6D)	5′-GAATGGCTGGCAATGGCG-3′	
3D2/6D_RT (make 6D)	5′-TAGCAGGATCAGTTCCCCTATCGCTGCCTGGATCTGAATTTCTTGAACTGTTGCGACTAC-3′	
3D2/6D_RS (make 6D)	5′-TTGAACTGTTGCGACTAC-3′	

Italicized bases: T7 promoter sequence

Table S2. Salt titration average $K_{d(1M)}$ and Z_{eff} values

	HIV-1 Gag		SARS-CoV-2 WT Np		SARS-CoV-2 3xD1 Np	
	$K_{d(1M)}$ (M)	Z_{eff}	$K_{d(1M)}$ (M)	Z_{eff}	$K_{d(1M)}$ (M)	Z_{eff}
SL1-5	$(2.1 \pm 1.8) \cdot 10^{-2}$	8.3 ± 0.9	$(3.6 \pm 1.4) \cdot 10^{-5}$	4.4 ± 0.3	$(5.9 \pm 4.4) \cdot 10^{-2}$	8.3 ± 0.9
SL1-4	$(1.2 \pm 1.7) \cdot 10^{-1}$	7.5 ± 1.6	$(2.6 \pm 2.7) \cdot 10^{-4}$	5.6 ± 0.8	31 ± 48	13 ± 2.9
SL5	$(1.4 \pm 1.1) \cdot 10^{-4}$	4.9 ± 0.5	$(5.0 \pm 3.9) \cdot 10^{-4}$	4.7 ± 0.7	38 ± 57	12 ± 2.4
HIV-1 TARpA	*$(2.2 \pm 1) \cdot 10^{-2}$	*9.1 ± 0.3	$(2.6 \pm 2.5) \cdot 10^{-4}$	6.2 ± 0.9	3.1 ± 3.9	12 ± 1.0
HIV-1 Psi	*$(5.2 \pm 1) \cdot 10^{-5}$	*5.0 ± 0.2	$(3.8 \pm 3.9) \cdot 10^{-5}$	4.3 ± 1.2	$(1.8 \pm 2.7) \cdot 10^{-2}$	8.3 ± 1.3

	SARS-CoV-2 3xD2 Np		SARS-CoV-2 6xD Np	
	$K_{d(1M)}$ (M)	Z_{eff}	$K_{d(1M)}$ (M)	Z_{eff}
SL1-5	$(2.5 \pm 1.4) \cdot 10^{-5}$	3.8 ± 1.0	$(3.1 \pm 5.9) \cdot 10^{-2}$	6.9 ± 2.6
SL1-4	$(5.5 \pm 5.4) \cdot 10^{-5}$	4.4 ± 1.8	$(6.8 \pm 9.4) \cdot 10^{-4}$	4.8 ± 1.0
SL5	$(2.2 \pm 1.4) \cdot 10^{-3}$	7.2 ± 0.5	$(6.8 \pm 12) \cdot 10^{-3}$	4.4 ± 1.9
HIV-1 TARpA	0.41 ± 0.1	10 ± 0.4	17.2 ± 28	10.1 ± 1.9
HIV-1 Psi	$(2.9 \pm 3) \cdot 10^{-4}$	6.9 ± 1.9	50.7 ± 52	13.7 ± 1.4

Predominantly hydrophobic interaction

Predominantly electrostatic interaction

Purely electrostatic interaction

*Webb, J.A. *et al. RNA*, **2013**. doi: 10.1261/rna.038869.113

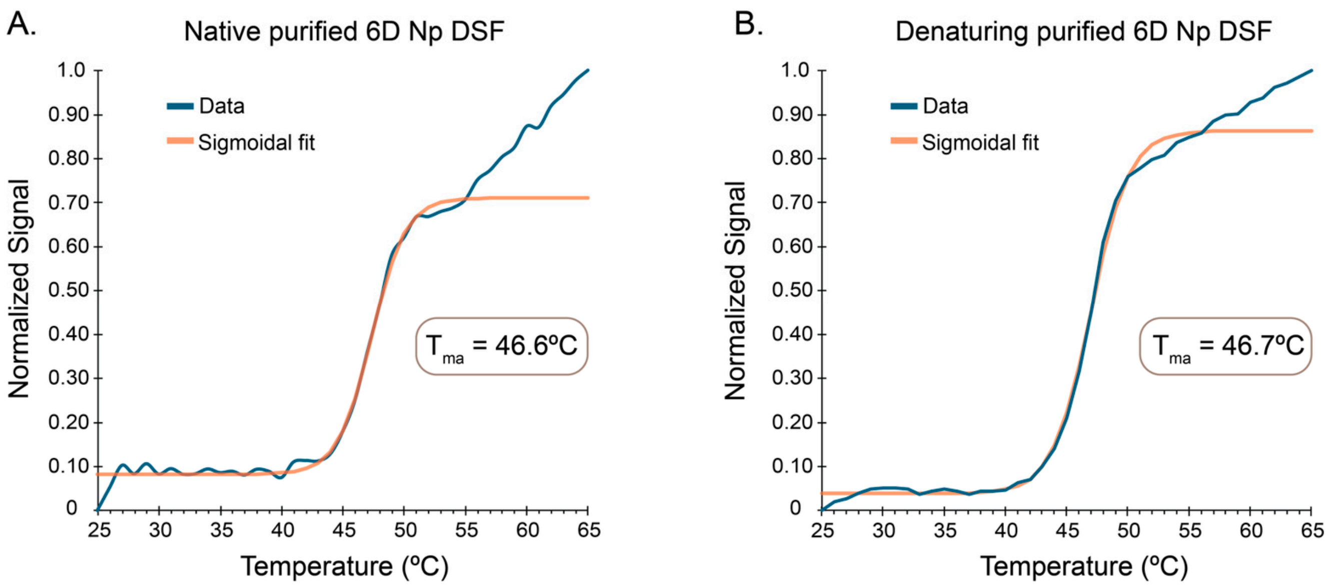

Figure S1. Representative DSF curves for natively purified 6D Np (A) and denaturing purified 6D Np (B) including the sigmoidal fit. Replicates shown in this figure are independent of the replicates used in Fig. 4 and were not used to determine average T_{ma} values, but the data collection and analysis methods are the same.

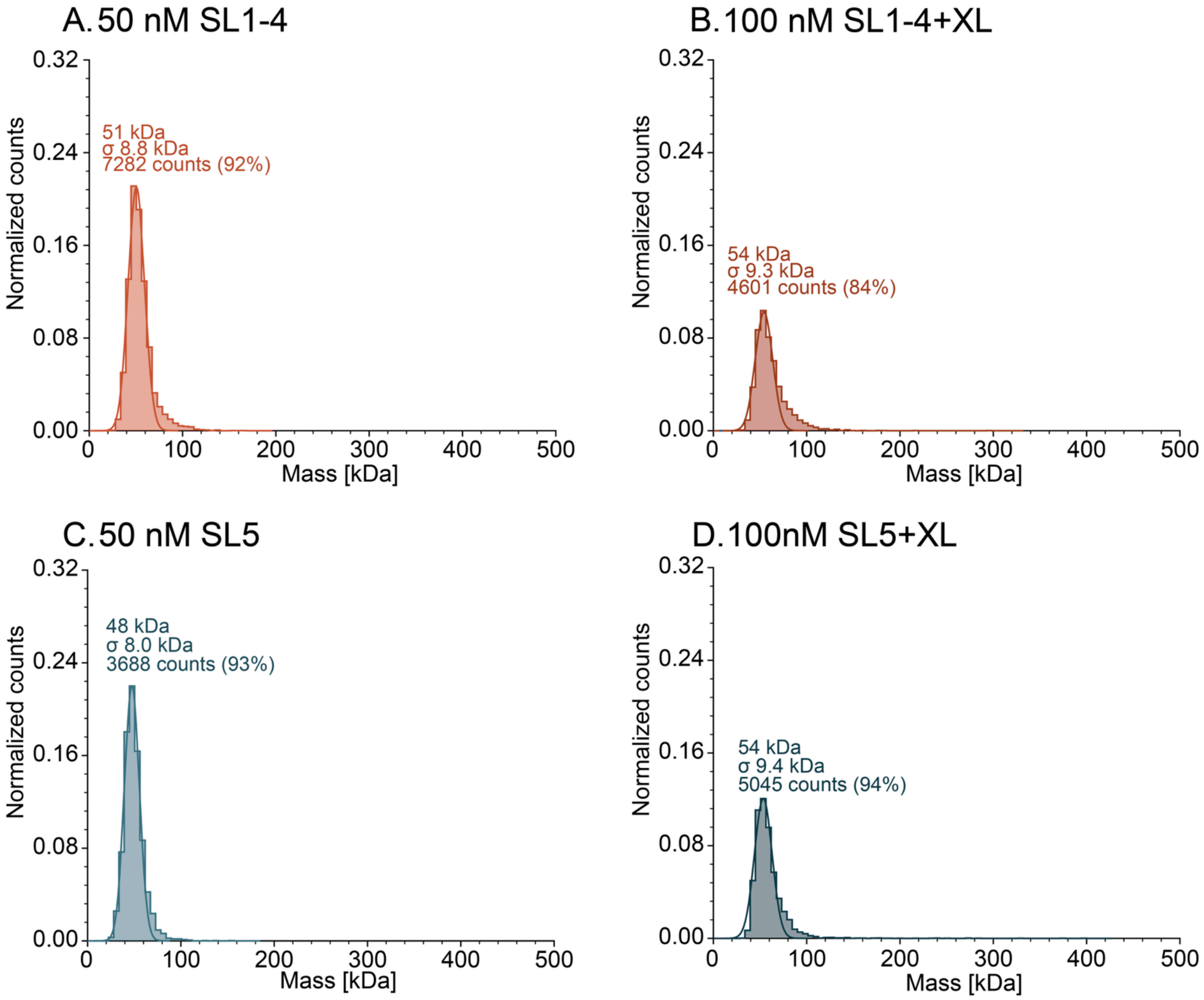

Figure S2. MP of SL1-4 and SL5 RNAs alone without (A,C) and with (B,D) crosslinker. The addition of crosslinker does not affect the overall mass distribution of either RNA.

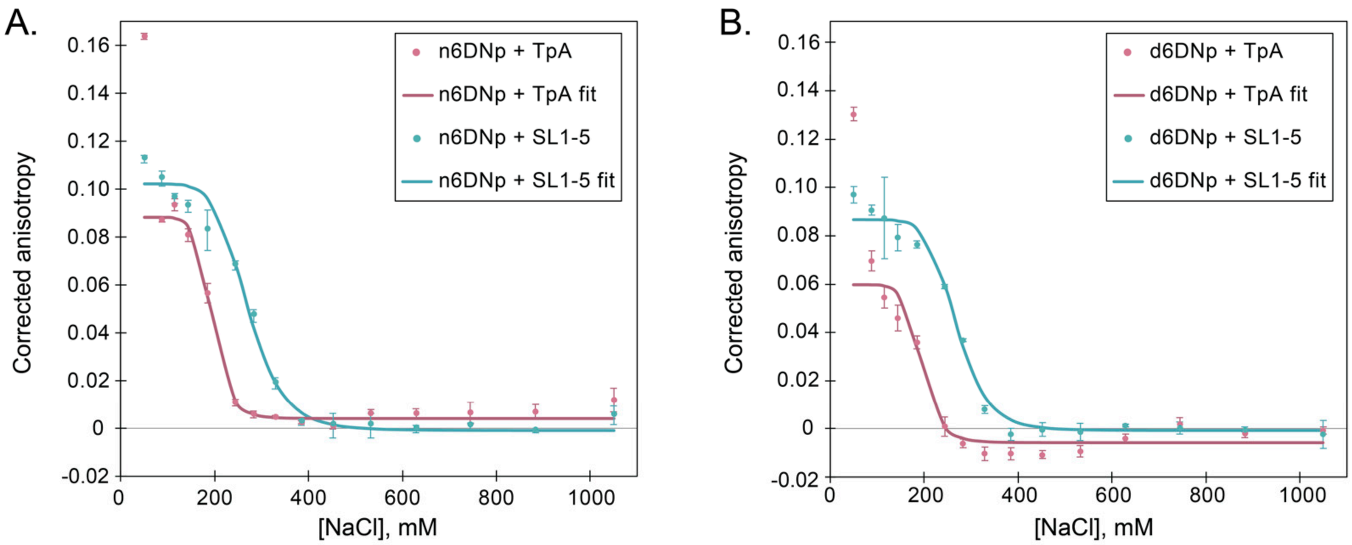

	native 6DNp		denaturing 6DNp	
	$K_{d(1M)}$	Z_{eff}	$K_{d(1M)}$	Z_{eff}
SL1-5	**5.18 * 10^{-3}**	**7.25**	**1.28 * 10^{-2}**	**8.24**
TARpolyA	**3.18**	**10.0**	**1.42**	**9.4**

Figure S3. Comparison of natively- and denaturing-purified 6D Np via salt-titration RNA binding assays. Anisotropy curves and fitted parameters are shown for both natively-purified 6D Np (A) and denaturing-purified 6D Np (B) binding to HIV-1 TARpolyA and SARS-CoV-2 SL1-5 RNAs. Both proteins' fitted values lie within the expected standard deviations displayed in Table S2.
